# Investigating socio-economic inequity in access to and expenditures on routine immunization services in Anambra state

**DOI:** 10.1186/s13104-017-2407-1

**Published:** 2017-02-01

**Authors:** Florence T. Sibeudu, Benjamin S. C. Uzochukwu, Obinna E. Onwujekwe

**Affiliations:** 1 Department of Nursing Science, NnamdiAzikiwe University, Nnewi Campus, Nnewi, Nigeria; 2 Department of Community Medicine, University of Nigeria, Enugu Campus, Enugu, Nigeria; 3 Health Policy Research Group, Department of Pharmacology and Therapeutics, University of Nigeria, Enugu Campus, Enugu, Nigeria; 4 Department of Health Administration and Management, University of Nigeria, Enugu Campus, Enugu, Nigeria

**Keywords:** Socio-economic status, SES, Access, Utilization, Inequity, Routine immunization, Expenditures

## Abstract

**Background:**

Addressing existing inequities in the utilization of priority health services such as routine immunization is a current public health priority. Increasing access to routine immunization from the current low levels amongst all socio-economic status groups in Nigeria is challenging. However, little is known on the level of SES inequity in utilization of routine immunization services and such information which will inform the development of strategies for ensuring equitable provision of routine immunization services in the country. The study was a cross sectional household survey, which was undertaken in two randomly selected communities in Anambra State, southeast Nigeria. A pre-tested interviewer administered questionnaire was used to collect data on levels of access to RI by children under-2 years from randomly selected households. In each household, data was collected from the primary care givers or their representative (in their absence). The relationship between access to routine immunization and socio-economic status of households and other key variables was explored in data analysis.

**Result:**

Households from high socio-economic status (well-off) groups utilized routine immunization services more than those that belong to low socio-economic status (poor) groups (X^2^ = 9.97, p < 0.002). It was found that higher percentage of low socio-economic status households compared to the high socio-economic status households received routine immunization services at public health facilities. Households that belong to low socio-economic status groups had to travel longer distance to get to health facilities consequently incurring some transportation cost. The mean expenditures on service charge for routine immunization services (mostly informal payments) and transportation were US$1.84 and US$1.27 respectively. Logistic regression showed that access to routine immunization was positively related to socio-economic status and negatively related to distant of a household to a health facility.

**Conclusion:**

Ability to pay affects access to services, even when such services are free at point of consumption with lower socio-economic status groups having less access to services and also having other constraints such as transportation. Hence, innovative provision methods that will bring routine immunization services closer to the people and eliminate all formal and informal user fees for routine immunization will help to increase and improve equitable coverage with routine immunization services.

**Electronic supplementary material:**

The online version of this article (doi:10.1186/s13104-017-2407-1) contains supplementary material, which is available to authorized users.

## Background

In Nigeria, there are low levels and inequitable access to and utilization of routine immunization (RI) services [1]. This is despite the fact that immunization services are provided free of charge to consumers [2]. Addressing inequities in access and utilization of health services such as RI has been a priority challenge in the health sector [3–5]. In order to equitably improve the health of the population, everybody irrespective of his or her location and socio-economic status should have equal access to the health care services that they need [6]. In fact, socio-economic status (SES) has been identified as one of the major factors that influence health seeking behavior in the utilization of health care services [4]. SES is commonly conceptualized as the social standing or class of an individual or group often measured as a combination of education, income and occupation [7].

The Nigerian government stipulates that all children less than 1 year should receive all vaccines on the routine immunization schedule free of charge in both public and private health facilities [2]. To further ensure optimal utilization of immunization services, the Nigerian Government developed the ‘Reach Every District (RED)’ strategy [8, 9]. Consequently, the government established more primary health facilities so that each ward has at least a health post to be able to provide basic health services including immunization [10]. This was aimed to eliminate some of the barriers to access such as the distance of households to health facilities.

Notwithstanding, there is low coverage with immunization as only 25% of children age 12–23 months are fully vaccinated with BCG, measles, and three doses each of DPT and polio vaccines [11]. Consequently, vaccine preventable diseases contribute significantly to the death of children especially those less than 5 years [12].It accounted for approximately 22% of childhood deaths, amounting to over 200,000 deaths per year [13]. It is worthy of note that studies have identified and listed key challenges and bottlenecks impacting immunization performance in Nigeria some of which are the weak health and primary health care (PHC) system in most parts of the country, the poor engagement of the Community, missed opportunities for immunization and reaching under-served and hard-to-reach Communities in the routine immunization [14–17]. However,it has been found that there is low availability of information on the level of inequity in access to and utilization of RI in Nigeria and in many developing countries. Hence, leaving us with paucity of evidence on the factors that explain household access and utilization of RI services.

It is not clear how improvements in access to and utilization of routine immunization are distributed across different SES groups because of paucity of information on the issue. Broadly, two Israeli studies [18, 19] found that there was no relationship between socio-economic status and utilization of health services. However, studies from Cameroon [20], Zimbabwe [21], Tanzania [22] and Nigeria [6] found that SES was one of the key determinants of level of utilization of health services. Two studies from United States of America found that the major factors militating utilization of childhood immunization were clinic waiting time [23] and having more than two children in a household [24] showing that the finding is context specific. The 2008 Nigerian National Demographic Health Survey showed that migration [25], and socio-economic status [26, 27] influenced childhood immunization. A study from south–west, Nigeria found that cost of payment and transportation influenced utilization of RI [28].

Nigeria’s efforts at reducing the burden from vaccine preventable diseases have been constrained because of low coverage, especially of routine immunization. It had been claimed that the low levels of coverage are potentially due to inequities in access with the poorest households not having access to immunization services. Therefore, in order to solve the problems and increase coverage to immunization services, empirical evidence is needed in factors that affect coverage and the role of socioeconomic status in access to immunization services in Nigeria. The study provides new information on factors that determine the coverage of immunization amongst different population groups. The information is important to aid evidence-based decisions on development of strategies for equitable provision of immunization services in the country. This was by investigating the levels of access and utilization of RI services amongst different socio-economic status (SES) groups. In addition, it explored the influence of other factors on access to RI. This was in recognition that there is paucity of evidence on household factors that determine access to and utilization of RI services in many sub-Saharan African countries such as Nigeria.

## Study methods

The study was undertaken in two communities in Anambra State, southeast Nigeria. The state was chosen purposively as the base of the research team so as to provide an evidence of the effect of socio-economic status on access to routine immunization. The two communities that were used for the study were an urban community (Awada) and a rural community (Ezi-owelle). Anambra state has a population of 4,612,666 (projected from 2006 national census).The inhabitants of the two communities are mainly of the Igbo ethnic origin and are predominantly Christian. Anambra state is made up of 21 local government areas (LGAs). The data was collected from April through July 2011.

### Brief description of the health system and system of provision of routine immunization activities in Anambra State

The Anambra State Health system is regulated and managed by the State Ministry of Health (MOH). The health system constitutes of both public and private providers and they provide primary, secondary and tertiary health care services. The privately owned health facilities, which are for-profit organizations, comprise approximately 73% of all health facilities in the state.

Routine immunization (RI) services are provided by both public and private providers. Both groups of providers are all expected to provide RI services free of charge as stipulated by the National Policy on Immunization. The MOH has a public–private partnership (PPP) contract with many private facilities for the provision of routine immunization services.

In the state, the state immunization office at the state capital has the responsibility to receive and store vaccines and materials for immunization. The state office then distributes the supplies to the LGAs immunization offices. The LGA immunization office stores vaccines and materials for immunization in the LGA and it in turn distribute the vaccines to public and private health facilities within the LGA. On each immunization day, most health facilities visit the LGA immunization office for collection of vaccines and materials for immunization. At the next immunization day, the health facility returns used vaccine vials. Although the services are expected to be provided free of charge, some private and public health facilities charge consumers, in many cases, albeit informally for services.

### Data collection

A multistage sampling method was used to select the respondents. Firstly, simple random sampling method was used to select one local government area (LGA) from a sampling frame of a list of all the LGAs in Anambra State. Then, stratified sampling method was used to group the communities in the selected LGA into urban and rural communities. Then, using simple random sampling, one rural community and one urban community were selected from the sampling frame of rural and urban communities. The minimum sample size for the study was calculated using 72% state routine immunization coverage as the prevalence rate at 95% confidence interval giving a sample size of 310. This number was increased to 350 to cover for a 10% non-response rate. Proportionate sampling based on the population of children under the age of one year in the two communities was used to allocate the sample size. In the rural community the sample size was 150 households while in the urban community, the sample size was 200 households.

A pre-tested questionnaire (Additional file 1) was administered by trained interviewer to respondents. Using the primary health care (PHC) house numbering system as the sampling frame, households that had at least a child aged less than 2 years were randomly selected. In each household, one female household primary care giver (mother), or the representative (in the absence of the mother) was interviewed. Data was collected on households’ socio-economic and demographic characteristics; access and utilization of routine immunization; expenditures on RI; distance to the health facility for RI; transportation to the health facility for RI; household assets ownership amongst others.

### Data analysis

Cross-tabulations were utilized to examine bivariate associations between SES and immunization. Chi square was used to calculate p values. In addition, logistic regression analysis was undertaken with utilization of immunization as the dependent variable. The independent variables were: socio-economic status, geographic location, distance of health facility, payment of services and years of formal education of household primary care givers. There was a test for multicollinearity to examine the level of correlation amongst the independent variables. A full modelling approach was used to retain all the original variables in the logistic model. Variables were statistically significant if the p value is less than 0.05. In order to examine SES differences, principal component analysis was used to develop a socio-economic status index using information on household asset ownership and cost of food as undertaken in previous studies [29, 30]. The SES index was broken down into quartiles, namely: Q1 = most poor; Q2 = poor; Q3 = average; Q4 = rich.

## Results

The results show that 338 households participated in the study, which was a 97% response rate. The majority of the respondents were mothers (81%) (Table 1). Almost all the mothers were married with an average age of approximately 31 years. The table also shows that 95.3% of the respondents had some formal education.

**Table 1:** Socio-demographic characteristics of households

Variables	N = 338 n (%)
Marital status	
Married	324 (95.9)
Single	14 (4.1)
Age: Mean (SD)	30.9 (9.0)
Any formal education:	322 (95.3)
Mothers level of education:	
Primary	59 (17.5)
Secondary	182 (53.8)
Tertiary	81 (24.0)
None	16 (4.7)
Education of household heads	
Primary	77 (22.8)
Secondary	203 (60.1)
Tertiary	47 (13.9)
None	11 (3.2)
Status of the respondent	
Mother	275 (81.4)
Representative	63 (18.6)
Mother’s occupation	
Farmer	34 (10.1)
Unemployed	86 (25.4)
Petty trader	93 (27.5)
Civil servant	24 (7.1)
Private employee	31 (9.2)
Big business	31 (9.2)
Self employed	39 (11.5)
Household head occupation	
Farmer	43 (12.7)
Unemployed	8 (2.4)
Petty trading	29 (8.6)
Civil servant	20 (5.9)
Private employee	17 (5.0)
Big business	151 (44.7)
Self employed	70 (20.7)

Table 2 shows that households’ from the higher socio-economic status (SES) group utilized routine immunization services more than those that belong the lower SES group. However, greater percentage of households that belong to the lower SES groups received routine immunization services in public health facilities, whilst greater percentage of households that belong to the higher SES groups received the services in private health facilities as shown by the Q1:Q4 ratio.

**Table 2:** Utilization of routine immunization by socio-economic status (SES) groups

Variables	Q1 most poor N = 85 n (%)	Q2 poor N = 84 n (%)	Q3 average N = 85 n (%)	Q4 rich N = 84 n (%)	Q1:Q4 ratio	N = 338 n (%)	X^2^ (p value)
Utilized routine immunization	66 (77.6)	71 (84.5)	80 (94.1)	77 (91.7)	0.86	294 (87.0)	9.97 (0.002)
Type of health facility attended							
Public	62 (72.9)	53 (63.1)	41 (48.2)	48 (57.1)	1.29	204 (60.4)	6.87 (0.009)
Private	23 (27.1)	31 (36.9)	44 (51.8)	36 (42.9)	0.63	134 (39.6)	
Waiting time (min)							
<15	39 (45.9)	40 (47.6)	19 (22.4)	23 (27.4)	1.70	121 (35.8)	11.97 (0.001)
15–39	24 (28.2)	20 (23.8)	30 (35.3)	25 (29.8)	0.96	99 (29.3)	0.53 (0.47)
40–60	8 (9.4)	5 (6.0)	7 (8.2)	8 (9.5)	1	28 (8.3)	0.01 (0.92)
>60	14 (16.5)	19 (22.6)	29 (34.1)	28 (26.7)	0.5	90 (26.6)	8.33 (0.004)

Table 2 also shows that the SES differences in utilization of immunization by type of health facility attended and waiting time were statistically significant. The table also shows from the Q1:Q4 ratio and figures that the lower SES groups waited longer in facilities to receive RI services when compared to the higher SES groups.

Table 3 shows that households that belong to the higher socio-economic group resided closer to the health facilities compared to households from the lower SES groups (p < 0.05). The table also shows that the lower SES groups mostly trekked to the health facilities, whilst the higher SES groups mostly used motorized transport to access the immunization services.

**Table 3:** Distance of health facility from household and means of transportation to health facility

Variables	Q1 most poor N = 85 n (%)	Q2 poor N = 84 n (%)	Q3 average N = 85 n (%)	Q4 rich N = 84 n (%)	Q1:Q4	X^2^ (p value**)**
Distance of health facility (km)						
<5	27 (31.8)	25 (29.8)	39 (45.9)	36 (42.9)	0.75	4.385 (0.036)
5–9	33 (38.8)	41 (48.8)	30 (35.3)	29 (34.5)	1.14	1.220 (0.269)
10–15	21 (24.7)	11 (13.1)	14 (16.5)	13 (15.5)	1.62	1.746 (0.186)
>15	4 (4.7)	7 (8.3)	2 (2.4)	6 (7.1)	0.67	0.024 (0.878)
Usual means of transport						
Trekking	47 (55.3)	42 (50.0)	43 (50.6)	39 (46.4)	1.21	1.141 (0.285)
Commercial bus	3 (3.5)	9 (10.7)	5 (5.9)	9 (10.7)	0.33	1.665 (0.197)
Motor cycle	31 (36.5)	31 (36.9)	32 (37.6)	23 (27.4)	1.35	1.297 (0.254)
Taxi	1 (1.2)	0 (0.0)	1 (1.2)	1 (1.2)	1	0.069 (0.792)
Free ride	3 (3.5)	1 (1.2)	4 (4.7)	1 (1.2)	3	0.198 (0.657)
Private car	0 (0.0)	1 (1.2)	0 (0.0)	11 (13.1)	0	17.721 (0.00003)

Table 4 shows that a greater percentage of households that belong to the higher SES group paid for routine immunization services when compared to households from the lower SES group. However, there were no statistically significant differences (p > 0.05) in payments for direct RI services and transportation by different SES groups.

**Table 4:** Payment and expenditures on routine immunization by SES

Variable	Q1 N = 85	Q2 N = 84	Q3 N = 85	Q4 N = 84	Q1:Q4 ratio	Total N = 338	Kruskal–Wallis test X^2^ (p value)
Paid for immunization: n (%)	43 (50.6)	64 (76.2)	73 (85.9)	68 (81.0)	0.63	248 (74.4)	32.11 (0.00)
Expenditure on services: US$ mean (SD)	1.76 (1.44)	1.60 (1.21)	2.01 (1.20)	1.93 (1.17)	0.87	1.84 (1.23)	6.75 (0.08)
Expenditure on transportation: US$ mean (SD)	1.29 (0.59)	1.17 (0.28)	1.13 (0.38)	1.53 (0.70)	0.85	1.27 (0.51)	0.80 (0.85)

The result of the multiple logistic regression analysis is presented in Table 5. The findings show that access to routine immunization was statistically significantly related to two variables, which were SES and the location of the facility (p < 0.05). However, whilst access was positively related to SES, it was negatively related to location of the health facility. The overall logistic model was statistically significant (p < 0.001). There was no evidence of multicollinearity in the regression model.

**Table 5:** Logistic regression analysis of access to routine immunization

Variables	Coefficient	Standard error	p value
Constant	0.926	0.113	0.000
Geographic location	−0.061	0.026	0.018
Distance	−0.009	0.022	0.697
SES	0.045	0.019	0.018
Years of formal education	−0.010	0.006	0.082
Payment of service	0.081	0.051	0.109

## Discussion

This study shows that SES plays an important role in determining utilization of routine immunization services. This is consistent with findings from similar studies done in other countries [31–34]. Also, as have been found in other studies that examined access to services, distance to a health facility is a significant predictor of use of services [5, 35]. However, although having education was positively related to access to routine immunization services, the relationship was not statistically significant.

In this study, households that belong to the higher SES groups had the greatest access to routine immunization services, whilst the lower SES group had the least access to the services. This may be because greater percentage of the higher SES groups lived nearer the health facilities and had better transportation services to the health facilities. This view is strengthened by the finding that the lower SES groups more than the other SES groups trekked to the health facilities to receive services. This could serve as constraining factor to access to services to the households, especially to the low SES group.

The evidence that households that belong to the “lower” SES group had the least level of access to routine immunization services was not expected given that the services are free at the public facilities where they mostly received services. However, our findings is consistent with those from similar studies in Nigeria [30, 36] and other countries [37–39] where high SES was identified as determinant of complete immunization but inconsistent with another study that reported that low socio-economic status was associated with high immunization completion rate [40].

The finding that health facilities were more proximal to household belonging to the high SES groups is potentially another reason why they had greater access to routine immunization compared to the low SES groups. This is supported from the findings of similar studies in Nigeria [41] and elsewhere Nigeria [42] where distance was identified as the major determinant of utilization of health care services including immunization services. However, another study in an urban area of a developed country found that distance is not a determinant for utilization of routine immunization [43]. The resort to trekking to facilities from their distal locations by majority of the lower SES would have further added as a cost and disincentive to access routine immunization services from health facilities.

There was inequity in type of facilities that were used with the higher SES groups having the highest utilization of services at private health facilities. This is a possible indication that there may be better quality services in private health facilities where services are not free, which informed high level of utilization of routine immunization among those that preferred that had higher levels of ability to pay. This finding is buttressed from evidence from a study in Uganda [44] where despite government removal of user fees in public health facility, majority of the people still preferred private health facilities. This phenomenon was attributed to better attitude of healthcare workers in private health facilities compared to those in public health facilities [45]. The public sector is usually characterized by long waiting time, which can negatively affect immunization coverage [46]. This study showed that indeed, the most poor and poor that mostly used public facilities actually had higher waiting times than the average and rich households that mostly used private facilities.

The finding that majority of the lower SES group paid for routine immunization services is a possible function of the fact that most of them received RI services from the private sector, which is known for profit maximization. Hence, although the national policy stipulates free provision of RI, some profit maximizing health providers in the private sector could have decided to charge sundry fees. However, such fees that were also paid in the public health facilities could be termed informal payments, since the providers are not supposed to charge fees in all circumstances. The incidence of informal payments as one of the challenges for optimal utilization of health services has been described in previous studies [47, 48]. Charging of fees, whether formal or informal can constrain access to services to poor people, especially in the widely available private sector and low level public sector. Other studies have shown that consumer expenditures on immunization services predicts levels of utilization of services [28, 49]. Payments by the “lower” SES group can lead to their incurring catastrophic health expenditures [44]. Therefore government should track and ensure that health workers in both private and public health facility do not charge fees for RI services.

The lack of differences in the expenditures on routine immunization and transportation across different SES groups is retrogressive as the poor are by implication devoting more of the percentage of their income to health expenditures that can further deter them from utilizing RI services. This may lead to some lower SES groups incurring catastrophic health expenditures because of such payments [50].

The inequity in access to services is contributed to by the finding that distance to health facilities and consequent transportation costs possibly limit access to RI services by the lower SES groups. Other studies also indentified transportation as a barrier to utilization of routine immunization in Nigeria [40, 46]. Hence, the expenditures for accessing RI services were high for the lower SES group, which poses a huge challenge to scaled-up delivery and utilization of RI services.

Some limitations of the study included the fact that most of the interviewers were made to visit some household several times before they could see the primary care givers or their representatives at home for data collection. The revisits increased the resources that were required to collect data.

## Conclusion

In conclusion, the study shows that ability to pay affects access to services, even when such services are free at point of consumption since the SES of a household influences utilization of routine immunization services, with lower SES groups having less access to services and also having other constraints such as transportation. It is important that government reviews public private partnership arrangement with private health facilities for routine immunization services with a view to consider reimbursement package for private health institution that offer routine immunization services to ensure that households receive immunization free of charge in all health facilities. In addition, the fact that low SES groups mostly received RI services at public health facilities, where they were also made to pay for the services, possibly further hindered equitable access to RI. Hence, government and its partners should innovate the delivery methods that will bring RI services closer to the people and institute monitoring mechanism to eliminate all formal and informal user fees for RI will help to increase and improve equitable coverage with RI services. Health care workers may also visit households to offer routine immunization services since distance of health facilities is a challenge for utilization of immunization.

## Additional file

**Additional file 1.** English-language version of the questionnaire. Routine Immunization utilization questionnaire. The questionnaire has three sections; Section A, B and C. Section A was designed to elicit data on socio-demographic characteristics, Section B to elicit data on household routine immunization utilization while Section C is for household assets ownership data.

## Abbreviations

RI: routine immunization
SES: socio-economic status
RED: reach every district
PHC: primary health care
