# Supplementary material for: Investigating socio-economic inequity in access to and expenditures on routine immunization services in Anambra state
Source: BMC Res Notes. 2017 Feb 1;10:78. doi: 10.1186/s13104-017-2407-1 (PMC5286773; doi:10.1186/s13104-017-2407-1)
Supplement: Supplementary file 1 — Additional file 1. English-language version of the questionnaire. Routine Immunization utilization questionnaire. The questionnaire has three sections; Section A, B and C. Section A was designed to elicit data on socio-demographic characteristics, Section B to elicit data on household routine immunization utilization while Section C is for household assets ownership data. [file 13104_2017_2407_MOESM1_ESM.docx]

**Routine Immunization utilization questionnaire**

This survey is intended to identify factors that affect access to routine immunization services and which group are much hit. Please answer to the best of your capability since your responses will influence how the government will respond to the improvement of immunization services in this area to promote access. Your participation is completely voluntary, and you do not have to answer any questions you do not want to answer.

**SECTION A: Socio-Demographic characteristics**

1. How old is the respondent? [ ] years

2. Respondent’s sex [ ] 1 = male 0 = female

3. What is your religion? 1=Yes 0=no

3a.Christianity [ ]

3b.Muslim [ ]

3c.Traditon [ ]

3d.Others [ ] (Please Specify)……………………

4. What is your denomination? 1=yes 0=no

4a.Anglican [ ]

4b.Roman Catholic [ ]

4c.Penticostal [ ]

4d.Others [ ] (please specify)……………………..

5. Marital status 1 = yes 0 = no

5a. Single [ ]

5b. Married [ ]

6. Relationship between respondent and child that received immunization 1 = yes 0 = no

6a. Mother [ ]

6b. Father [ ]

6c. Grandmother [ ]

6d. Grandfather [ ]

6e. Brother/Sister [ ]

6f. Other [ ] (Please specify)_________________________________

7. Do you or the mother (if the respondent is not the mother) had any formal education? [ ] 1 = yes (GO TO QUESTION 8) 0 = no (GO TO QUESTION 10)

8. How many years of formal education [ ] years*

9. What was the highest completed education level? 1 =yes 0 *= no

9a. Still in primary school [ ]

9b. Primary [ ]

9c. Junior secondary [ ]

9d. Senior secondary [ ]

9e. University [ ]

9f. Polytechnic [ ]

9g. Other [ ] Please specify________________________________________

10.What is the occupation of the respondent or mother (if the respondent is not the mother)? ? 1 = yes 0 = no

10a. Farmer [ ]

10b. Unemployed [ ]

10c. Petty trading [ ]

10d. Government worker [ ]

10e. Employed in private sector [ ]

10f. Big business [ ]

10g. Self-employed professional [ ]

10h. Others [ ] Please specify_______________________

11. What occupation is the household head’s major source of getting

money or your occupation if you are the household head?1=yes; 0 = no

11a. Farmer [ ]

11b. Unemployed [ ]

11c. Petty trading [ ]

11d. Government worker [ ]

11e. Employed in private sector [ ]

11f. Big business [ ]

11g. Self-employed professional [ ]

11h. Others [ ] Please specify_______________________

12. What is the household’s head’s level of education? (Enumerator: Ask if

the respondent is not the household head)

12a. Primary [ ]

12b. Junior secondary [ ]

12c. Senior secondary [ ]

12d. University [ ]

12e. Polytechnic [ ]

12f. Other [ ] Please specify____________________________

13. How many people are there in your household? [ ]

**SECTION B: Immunization utilization data**

14.Did you go for routine immunization services? YES=1 No =0 [ ]

15. The last immunization that was received by the child , was it received at

the actual age the child is supposed to receive it [ ] 1 = yes (IF YES,

GO TO QUESTION 17) 0 = no (IF NO, GO TO QUESTION 16)

16. Why did the child not receive the immunization at the correct age? 1 =

yes 0 = no

a. Child was sick [ ]

b. No money to take child to the health center [ ]

c. did not remember the time for the next dose [ ]

d. Did not understand the schedule of immunization [ ]

e. My child reacted to previous immunization [ ]

f. My job does not allow me [ ]

g. Didn’t think it was necessary [ ]

h. I came to the health center and there was no vaccine [

i. Health workers were on strike [ ]

j. The health center is too far from my home [ ]

k. No reason [ ]

l. Other [ ] Please specify_________________________

17. How old is the child receiving immunization [ ] months

18. What is the sex of the child receiving immunization [ ] 1 = male

0 = female

19.What type of health facility do you attend? 1=yes 0=no

a. Public [ ]

b. Private [ ]

20. What is the location of the health facility? 1 = yes 0 = no

a. urban area [ ]

b. Semi-urban area [ ]

c.Rural area [ ]

21. What is the distance from your house to health facility? 1=yes 0=no

a less than 5km[ ]

b.5-10km [ ]

c.10-15km [ ]

d. above 15km [ ]

22.How long did you wait in the health facility to actually be vaccinated

when you go for immunization? 1=yes 0=no

a.less than 15mins [ ]

b.15-30mins [ ]

c.30-60mins [ ]

d. above 1hr [ ]

23.Did you pay for the immunization service? 1=yes (if yes,go to question

Question 24) 0 = no (IF NO,GO TO QUESTION 25)

24. If yes, how much did you normally pay? Specify ………………

25. Has there been any time you were refused immunization at the health

center? [ ] 1 = yes (GO TO QUESTION 26) 0 = no (GO TO

QUESTION 27)

26. What was/were the reasons for the refusal? 1 = yes 0 = no

26a. Could not pay the cost of the immunization [ ]

26b. Did not come on time to the health center [ ]

26c. I quarreled with the health workers [ ]

26d. Other [ ] Please specify_________________________

27. What is your usual means of transport to health facility? 1 = yes 0 = no

27a. I walked [ ]

27b. Took a bus [ ]

27c. Took okada [ ]

27d. Took a taxi [ ]

27e. Free ride [ ]

27f. Private car [ ]

28. How much do you pay for transportation to health facility for

immunization? [________]naira

29. How much will you pay to go home? [_________]naira

30. Were you told when your next appointment will be? [ ] 1 = yes 0 =

no

31. Where did you hear of routine immunization first? 1 = yes 0 = no

31a. Health workers [ ]

31b. Church [ ]

31c. Friends and relations [ ]

31d. News paper [ ]

31e. Radio/TV announcements [ ]

31f. Town crierers [ ]

31g. Other [ ] Please specify

32. Have you heard of Acute flaccid paralysis surveillance (searching for children less than 15 years who suddenly developed weakness of the limbs)? [ ] 1 = yes (GO TO QUESTION 33) 0 = no (GO TO QUESTION 34)

33. What was/were your source/sources of information 1 = yes 0 = no

33a. Health workers [ ]

33b. Church [ ]

33c. Friends and relations [ ]

33d. News paper [ ]

33e. Radio/TV announcements [ ]

33f. Town crierers [ ]

33g. Other [ ] Please specify___________________

34. Have you heard of National Immunization Days(NIDs)? [ ] 1 = yes (GO TO QUESTION

35) 0=no (GO TO QUESTION 36)

35. What was/were your source/sources of information 1 = yes 0 = no

35a. Health workers [ ]

35b. Church [ ]

35c. Friends and relations [ ]

35d. News paper [ ]

35e. Radio/TV announcements [ ]

35f. Town crierers [ ]

35g. Other [ ] Please specify_________________________

36. How do you rate the services of the health workers in the health facility where you go for immunization services ? [ ] 1 = yes 0 = no

36a. Very poor [ ]

36b. Poor [ ]

36c. Good [ ]

36d. Very good [ ]

36e. Don’t know [ ]

**SECTION C: Household asset ownership data**

37. About how much did your household spend in the last one week on food items? [_________] naira

38. If the food you produced at home in the last one week but also consumed were bought, about how much would they have cost? [__________] naira

39. Does anyone in your household own any of the following? 1 = yes 0 = no

39a. Radio [ ]

39b. Television [ ]

39c. Air conditioner [ ]

39d. Bicycle [ ]

39e. Motorcycle [ ]

39f. Car [ ]

39g. Fridge [ ]

39h Generator [ ]

39i Electric fan [ ]

40. What is your household major source of water? 1 = yes 0 = no

40a. Private home tap [ ]

40b. Public pump [ ]

40c. Well [ ]

40d. Surface water e.g. stream, river, etc. [ ]

40e. Water tanker supplies water in home tank [ ]

40f. Rainwater [ ]

40g. Commercial bottled water [ ]

40h. Other [ ] please specify______________________

41. What kind of toilet facility do you have in your home? 1 = yes 0 = no

41a. Water cistern [ ]

41b. Private pit latrine [ ]

41c. Shared pit latrine [ ]

41d. No facility [ ]

41e. Other [ ] Please specify: ______________________________

42. How many persons sleep in a room in your house? [_____] persons

**THANK YOU**
